# Acrylamide coadministration modulates hepatic ROS-mediated apoptotic DNA damage and inflammation induced by TiO_2_ nanoparticles in mice

**DOI:** 10.1038/s41598-025-10915-0

**Published:** 2025-07-15

**Authors:** Hanan R.H. Mohamed, Clara Y. L. Azer, Ayman Diab, Gehan Safwat

**Affiliations:** 1 Zoology Department, Faculty of Science, Cairo University, Giza, Egypt; 2 Faculty of Biotechnology, October University for Modern Sciences and Arts, 6th October, Egypt

**Keywords:** Acrylamide, TiO_2_ nanoparticles, Concurrent exposure, hepatotoxicity, DNA damage, Inflammation and ROS generation, Genetics, Molecular biology, Natural hazards, Health care

## Abstract

The widespread human consumption of food and commercial products containing acrylamide and titanium dioxide (TiO_2_) nanoparticles highlights the need to assess the risks of their concurrent exposure. However, almost no studies have explored the effect of acrylamide and TiO_2_ nanoparticles co-exposure on genomic DNA integrity and inflammation induction in hepatic tissues. Consequently, this study aimed to estimate the impact of acrylamide and TiO_2_ nanoparticles coadministration on the genomic DNA integrity, reactive oxygen species (ROS) generation and expression level of apoptotic and inflammatory genes in mice hepatic tissues. Mice were orally administered acrylamide (3 mg/kg) or/and TiO_2_ nanoparticles (5 mg/kg) five times a week over two successive weeks. Genomic DNA integrity was assessed using alkaline Comet and Laddered DNA fragmentation assays, while ROS level was measured using 2, 7- Dichlorofluorescein diacetate dye. The expression level of inflammatory and apoptotic genes was quantified using quantitative real-time PCR (qRT-PCR). The results indicated that either acrylamide (3 mg/kg) or TiO_2_ nanoparticles (5 mg/kg) alone significantly disrupted DNA integrity, increased ROS level, and upregulated inflammatory (INOS, COX-2) and apoptotic (p53) gene expression, while downregulating the anti-inflammatory HO-1 gene. However, the coadministration of acrylamide and TiO_2_ nanoparticles resulted in even greater DNA damage, higher ROS production, and a further increase in inflammatory and apoptotic gene expression, along with a more pronounced decrease in HO-1 expression compared to the effects of either agent alone. In conclusion these findings suggest that chronic coadministration of acrylamide and TiO2 nanoparticles, even at low doses, amplifies the genomic DNA damage and inflammation induced by each agent individually, exacerbating hepatic cell stress. Therefore, avoiding simultaneous exposure to acrylamide and TiO2 nanoparticles is recommended to reduce the risk of severe toxic effects.

**Supplementary Information:**

The online version contains supplementary material available at 10.1038/s41598-025-10915-0.

## Introduction

The widespread use of nanoparticles, particularly titanium dioxide (TiO_2_), in food, cosmetics, plastics, and medical applications has raised concerns about human exposure and potential health risks^1,2^. Oral intake is a major route of exposure, with estimated daily consumption of TiO_2_ nanoparticles ranging from 5 to 50 mg through common products such as candy, toothpaste, preserved foods and drinking water^3,4^.

Orally ingested TiO_2_ nanoparticles have been shown to translocate through the bloodstream and accumulate in vital organs, including the liver, kidneys, stomach, and brain, leading to diverse toxicities such as hepatotoxicity, nephrotoxicity, gastrotoxicity, and neurotoxicity^1,3,5,6^. Notably, TiO_2_ nanoparticles have been shown to induce genotoxic effects, including chromosomal abnormalities, DNA damage, and alterations in apoptotic gene expression^3,5,7–9^. For instance, Mohamed et al.^1^ recently reported significant DNA damage and mutations in apoptotic genes within the hepatic tissues of mice orally exposed to TiO_2_ nanoparticles. However, these studies largely examine TiO_2_ in isolation, despite the reality that humans are often exposed to multiple environmental contaminants simultaneously.

One such co-exposure involves acrylamide, a toxic compound formed during the high-temperature cooking (> 120 °C) of carbohydrate- and protein-rich foods such as potatoes, bread, cakes, and chips. Levels of acrylamide in these foods typically range from 5 to 50 mg/kg, with drinking water containing approximately 0.5 mg/L^10–12^. Regular consumption of fried foods, baked goods, and beverages like coffee can therefore lead to substantial acrylamide exposure. The genotoxicity of acrylamide is well documented in both in vitro and in vivo studies. It interacts with nucleic acids and proteins, causing chromosomal aberrations, DNA damage, and mutations^13–16^. Oral administration of acrylamide has also been shown to increase micronuclei frequency and induce chromosomal aberrations in animal models^13–17^.

Given the high likelihood of concurrent human exposure to TiO_2_ nanoparticles and acrylamide through diet and environment, there is growing scientific interest in understanding the combined toxic effects of these agents. However, to date, very few studies have addressed their coadministration. Notably, only two recent studies have reported that combined exposure exacerbates genomic and mitochondrial DNA damage, mitochondrial dysfunction, oxidative stress, and inflammation in kidney and brain tissues of mice^16,18^. The impact of this combined exposure on hepatic genomic integrity and inflammatory responses remains largely unexplored.

Therefore, this study was conducted to estimate the effects of acrylamide (3 mg/kg b.w) coadministration on the genotoxicity, oxidative stress, and gene expression changes induced by TiO_2_ nanoparticles (5 mg/kg b.w) in mouse hepatic tissue. DNA integrity was assessed using alkaline Comet assay, reactive oxygen species (ROS) level were studied using 2’,7’-dichlorofluorescein diacetate (2,7-DCFH-DA) dye, and the expression of apoptotic and inflammatory genes was quantified using quantitative Real-Time PCR (qRT-PCR).

The chosen dose of TiO_2_ nanoparticles (5 mg/kg body weight) reflects estimated daily human exposure from dietary and consumer sources. According to studies and regulatory data, human intake of TiO_2_ nanoparticles can range from 1 to 5 mg/kg/day, particularly in children due to higher consumption of sweets, chewing gum, and processed foods containing TiO_2_ as a whitening agent (4). Therefore, 5 mg/kg is within the upper range of potential human exposure and has been widely used in in vivo studies to model realistic, sub-chronic oral exposure without causing acute toxicity. Similarly, the acrylamide dose of 3 mg/kg was selected to model dietary exposure levels that could occur under high consumption conditions. Although average human exposure is generally lower, occasional intake, especially in individuals consuming high amounts of fried or baked starchy foods (e.g., chips, bread, coffee), can result in significantly higher acrylamide intake. Previous toxicological studies have demonstrated that 3 mg/kg in rodents induces detectable genotoxic and oxidative stress responses while remaining below levels associated with systemic toxicity (11). Thus, both doses were chosen to simulate potential upper-bound chronic dietary exposures in humans, making the findings more relevant to real-world risk assessment and public health implications.

## Materials and methods

### Animals and ethical consideration

Male Swiss webster mice, weighing between 20 and 25 g and aged 10 to 12 weeks, were procured from the Animal House of the National Organization for Drug Control and Research (NODCAR). Prior to the commencement of treatment, the mice were allowed a one-week acclimatization period within the animal house environment of the Zoology Department, Faculty of Science at Cairo University. During this time, the mice were kept under standard dark/light cycles and provided with unrestricted access to a standard diet of pellets and water. The experimental design and procedures were thoroughly reviewed and received approval from the Institutional Animal Care and Use Committee (IACUC) at Cairo University, under approval number CU/I/F/15/18. This study adheres to the ARRIVE guidelines, ensuring transparency and reproducibility. Furthermore, all animal handling and experimental practices were conducted in accordance with the National Institutes of Health Guidelines for the care and use of laboratory animals.

### Chemicals

In this study, we utilized TiO_2_ nanoparticles and acrylamide sourced from Sigma-Aldrich Chemical Company located in St. Louis, MO, USA. The TiO_2_ nanoparticles, characterized by an average size of less than 100 nm, were carefully suspended in deionized distilled water immediately prior to their application. This procedure enabled the preparation of a TiO_2_ dosage set at 5 mg/kg body weight (b.w), aligning with the prescribed human exposure limit^4,19^. Concurrently, acrylamide was completely solubilized in deionized distilled water to formulate a test dose of 3 mg/kg b.w, which is also consistent with the human exposure guidelines^11^. All other chemicals and reagents utilized throughout the experiments were of analytical and molecular biology grade, ensuring the integrity and reliability of the results obtained.

### Characterization of TiO_2_ nanoparticles

Nano-TiO_2_ powders were sourced from Sigma-Aldrich Chemical Company (St. Louis, MO, USA), designated with a CAS number of 13463-67-7 and a reported purity of 99.5%. The characterization of these TiO_2_ nanoparticles was conducted in accordance with the methodology outlined by Safwat et al.^16^. X-ray diffraction (XRD) analysis confirmed the TiO_2_ crystal purity by revealing characteristic peaks at diffraction angles of 25.2°, 27.8°, 36.1°, 41.2°, and 54.7°. Transmission electron microscopy (TEM) of the aqueous TiO_2_ nanoparticle suspension showed polyhedral particles with uniform dispersion and an average size of approximately 60 nm.

### Study design

According to recently published studies^16,18^, a total of twenty-four male mice were randomly divided into four experimental groups, each consisting of six mice. The mice in the first group (Group I) served as a negative control and were orally administered distilled deionized water. In contrast, mice in the remaining groups (Groups II to IV) received either acrylamide alone at a dose of 3 mg/kg body weight^11^, TiO_2_ nanoparticles alone at a dose of 5 mg/kg body weight^4,19^, or a combination of both acrylamide and TiO_2_ nanoparticles administered simultaneously. These substances were administered five times per week over a period of two weeks. At the end of the treatment period, all mice were anesthetized using isoflurane inhalation to minimize discomfort, and subsequently euthanized by cervical dislocation 24 h after the final administration. Liver tissues were carefully dissected and immediately stored at − 80 °C for subsequent molecular analyses.

### Estimation of genomic instability

The influence of TiO_2_ nanoparticles (5 mg/kg b.w) or/and acrylamide (3 mg/kg b.w) multiple oral administration on the integrity of genomic DNA was studied by assessing the induction of genomic DNA damage in the hepatic tissues of the negative control group and TiO_2_ nanoparticles or/and acrylamide administered groups utilizing two key analytical methods: laddered DNA fragmentation and alkaline comet assays.

### Ladder DNA fragmentation assay

The ladder DNA fragmentation assay was carried out according to the protocol outlined by Sriram et al.^20^:Approximately 50 mg of hepatic tissue was carefully homogenized in a cold Tris-EDTA (TE) and sodium dodecyl sulfate (SDS) lysis buffer. The homogenized samples were incubated in the lysis buffer for one hour at 37 °C to facilitate cell lysis. After incubation, Proteinase K was added to all samples to degrade proteins, and the samples were further incubated at 50 °C. Cold absolute ethanol was added to the lysate to isolate genomic DNA. The precipitated genomic DNA was then dissolved in deionized distilled water for subsequent analysis. A total of 15 µl of the dissolved genomic DNA, containing approximately 3 µg of DNA, was loaded onto a 1% agarose gel. The samples were subjected to electrophoresis at 70 V. The gel was visualized and photographed using a UV trans-illuminator to assess the extent of DNA fragmentation.

### Alkaline comet assay

The assessment of genomic DNA breakages in hepatic tissues across the control group and mice orally given TiO_2_ nanoparticles (5 mg/kg b.w) or/and acrylamide (3 mg/kg b.w) was conducted utilizing the alkaline Comet assay, adapting the Tice protocol^21^ with minor modifications as outlined below. Hepatic tissues were gently minced, followed by the preparation of a cell suspension. Specifically, 10 µl of this cell suspension containing about 10,000 cells was combined with 75 µl of low melting agarose. The resultant mixture was promptly spread onto a fully frosted glass slide that was pre-coated with 1% normal melting agarose. After allowing the slide to dry, it was subjected to cold lysis using a buffer composed of 2.5 M NaCl, 100 mM EDTA, and 10 mM Tris (pH 10), to which freshly added 1% Triton X-100 and 10% DMSO were incorporated. This setup was incubated in the dark at 4ºC for duration of 24 h. Following this incubation period, the slides were pre-incubated in alkaline electrophoresis buffer for 15 min. The denatured genomic DNA was electrophoresed for 30 min at a voltage of 25 V and a current of 300 mA, corresponding to field strength of 0.90 V/cm. Post-electrophoresis, the slides underwent a neutralization process in Trizma base, were fixed in cold absolute ethanol, dried, and subsequently stored at room temperature. The re-annealed double-stranded DNA was stained with ethidium bromide. Examination and photography of the slides were performed using an epi-fluorescent microscope. For each sample, a total of fifty nuclei were analyzed employing TriTek Comet Score TM Freeware v1.5 scoring software. The parameters assessed included tail length, percentage of DNA in the tail, and tail moment, which served as indicators of DNA breakages within the hepatic cells.

### Measuring the expression level of p53, INOS, HO-1 and COX-2 genes

Quantitative Real-Time Polymerase Chain Reaction (qRT-PCR) was performed to assess the mRNA expression levels of the p53, INOS, HO-1, and COX-2 genes within the hepatic tissues of both the negative control and TiO_2_ nanoparticles (5 mg/kg b.w) or/and acrylamide (3 mg/kg b.w) administered groups. Total hepatic RNA was isolated following the manufacturer’s protocol for the GeneJET RNA Purification Kit (Thermo Scientific, USA). The isolated RNA was then reverse transcribed into complementary DNA (cDNA) utilizing the Revert Aid First Strand cDNA Synthesis Kit (Thermo Scientific, USA). For the quantification of the p53, INOS, HO-1, and COX-2 gene expressions, separate qRT-PCR assays were conducted for each gene. These assays employed SYBR Green Master Mix along with the previously designed primer sequences, which are detailed in Table 1^22–24^. The housekeeping gene β-actin was used as a reference to standardize the expression levels of the target genes. The fold change in expression levels of the analyzed genes was calculated using the comparative Ct (ΔΔCt) method. By following these procedures, we were able to reliably measure and compare the gene expression levels across the different experimental groups.

**Table 1:** Sequences of the used primers in qRT-PCR.

Gene	Strand	Sequence
**p53**	**Forward**	5′-ACCATCGGAGCAGCCCTCAT-3′
	**Reverse**	5′-TACTCTCCTCCCCTCAATAAG-3′
**INOS**	**Forward**	5′-CGGGCATTGCTCCCTTCCGAAAT-3′
	**Reverse**	5′-CTTCATGATAACGTTTCTGGCTCT-3
**HO-1**	**Forward**	5′-TGAAGGAGGCCACCAAGGAGG-3′
	**Reverse**	5′-AGAGGTCACCCAGGTAGCGGG-3′
**COX-2**	**Forward**	5′-ACCATTTGAACTATTCTACCAGC-3′
	**Reverse**	5′-AGTCGGCCTGGGATGGCATCAG-3
**β-actin**	**Forward**	5′-TCACCCACACTG TGCCCATCT ACG A-3′
	**Reverse**	5′-GGATGCCACAGGATTCCATACCCA-3′

### Studying the generation of intracellular ROS

Hepatic cells of negative control group and TiO_2_ nanoparticles (5 mg/kg b.w) or/and acrylamide (3 mg/kg b.w) administered mice were evaluated for ROS generation using 2, 7-dichlorofluorescin diacetate (DCFH-DA) dye^25^. A suspension of hepatic cells was prepared and mixed with the DCFH-DA dye. The resultant mixture was incubated in the dark for 30 min to facilitate the passive diffusion of the dye into the hepatic cells. Upon entering the cells, the dye reacts with ROS, yielding a highly fluorescent dichlorofluorescein compound. Following incubation, the cells were spread onto a clean microscope slide. The emitted fluorescent light was then visualized and captured using an epi-fluorescence microscope at a magnification of 200×. The captured photos were analyzed using Image analysis software and intensity of emitted fluorescence (indicator of ROS generation level) was expressed as mean ± SD.

### Statistical analysis

Data from the Comet assay and qRT-PCR were analyzed using the Statistical Package for the Social Sciences (SPSS), version 20, with a significance threshold set at *p* < 0.05. One-way analysis of variance (ANOVA) was employed, accompanied by Duncan’s test, to draw comparisons between the negative control group and the three treatment groups. All results are expressed as mean ± Standard Deviation (S.D).

## Results

### Disruption of genomic DNA integrity

The results of laddered DNA fragmentation and alkaline Comet assay revealed that the oral coadministration of acrylamide with TiO_2_ nanoparticles motivated TiO_2_ nanoparticles induced disruption of genomic DNA integrity as described below:

### Laddered DNA fragmentation

**Fig. 1:**
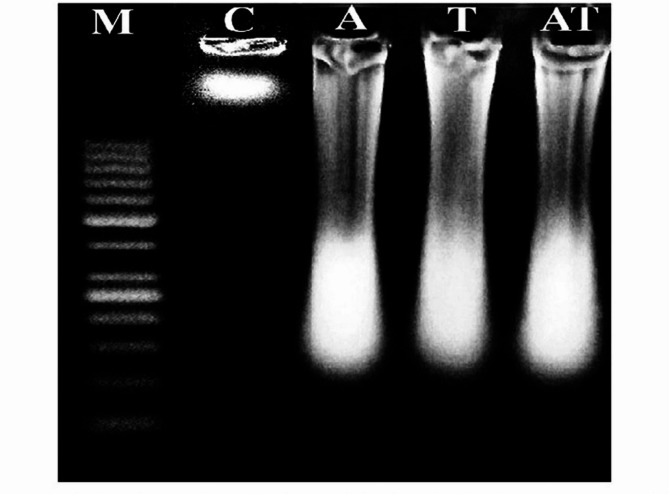
The electrophoresed pattern of genomic DNA extracted from the negative control mice (C) and mice administered acrylamide (A), TiO_2_NPs (T), or acrylamide with TiO_2_ nanoparticles (AT) on an ethidium bromide stained agarose gel. M: Marker.

As displayed in Fig. 1, and supplementary Fig. 1S oral administration of acrylamide (3 mg/kg bw) or TiO_2_ nanoparticles (5 mg/kg bw) for two successive weeks (five times per week) led to significant degradation of hepatic genomic DNA, as evident from the highly fragmented and smeared appearance of electrophoresed DNA on agarose gel, compared to the intact genomic DNA observed in the negative control group. Furthermore, simultaneous coadministration of acrylamide and TiO_2_ nanoparticles caused notably greater DNA degradation than that observed with acrylamide or TiO_2_ nanoparticles administered separately (Fig. 1).

### Alkaline comet assay

**Fig. 2:**
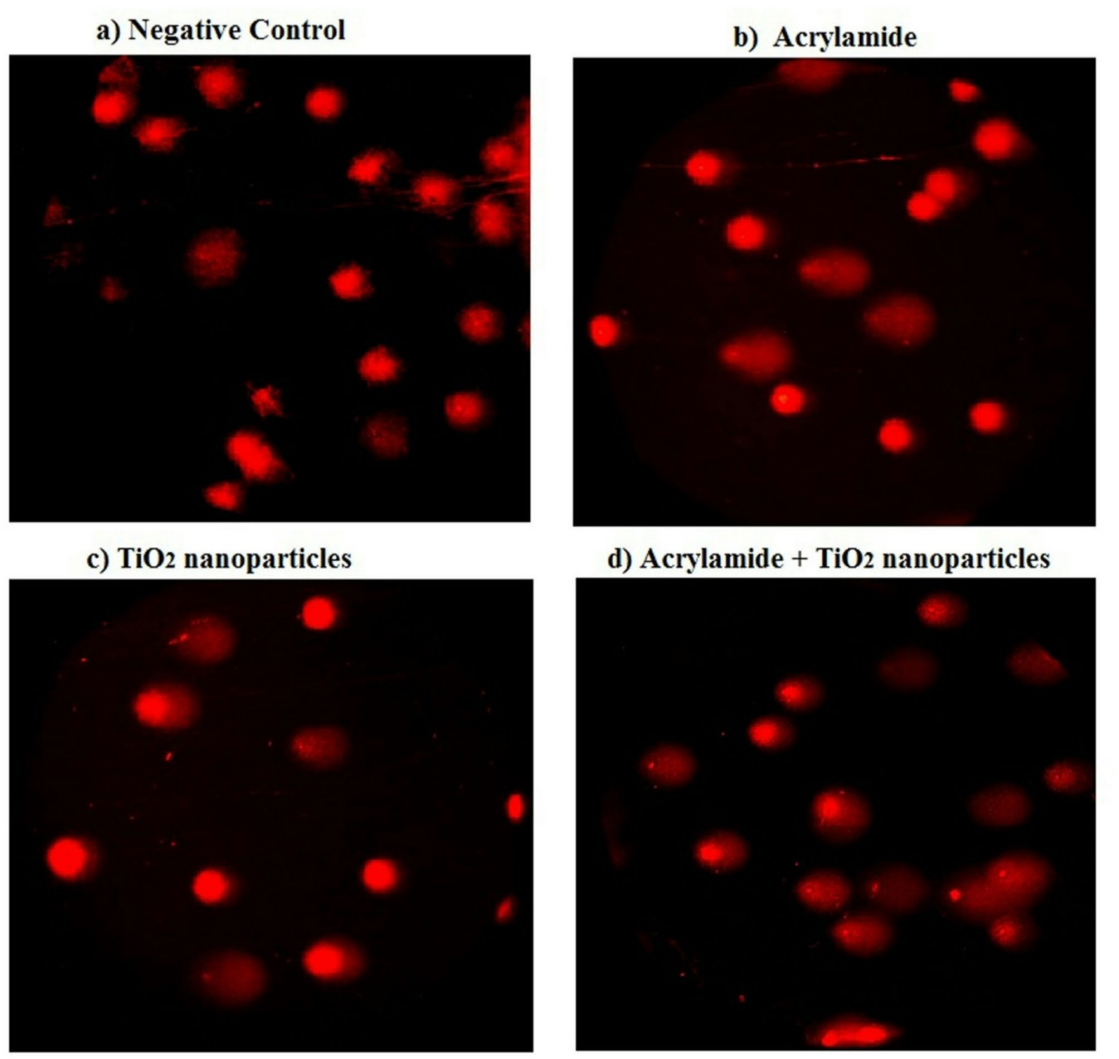
Representative photomicrograph for the observed comet nuclei in the hepatic tissues of **a**) negative control group, **b**) acrylamide administrated group, **c**) TiO_2_ nanoparticles administrated group, and **d**) group administered acrylamide with TiO_2_ nanoparticles.

Consistent with the laddered DNA fragmentation assay results, the alkaline Comet assay shown in Table 2; Fig. 2, revealed a significant (*p* < 0.001) increase in DNA damage induced by TiO_2_ nanoparticles following their simultaneous coadministration with acrylamide. This significant increase was evidenced by statistically significant elevations (*p* < 0.001) in DNA damage indicators including %DNA in the tail and tail moment, in the hepatic tissues of mice coadministered TiO_2_ nanoparticles and acrylamide compared to those administered either compound alone (Table 2). Additionally, notable increases (*p* < 0.001) in hepatic tail length, %DNA in the tail, and tail moment were observed following the separate administration of acrylamide or TiO_2_ nanoparticles for two weeks compared to the negative control group (Table 2).

**Table 2:** Induction of DNA damage in the hepatic tissues of the negative control group and groups given orally TiO_2_ nanoparticles (TiO_2_-NPs) or/and acrylamide.

Group	Treatment (dose mg/kg)	Tail length (px)	%DNA in tail	Tail moment
I	Negative control (0 mg/kg)	4.42 ± 0.87 ^a^	16.70 ± 1.15 ^a^	0.75 ± 0.18 ^a^
II	Acrylamide (3 mg/kg)	11.41 ± 0.53 ^b^	24.71 ± 0.98 ^b^	2.94 ± 0.30 ^b^
**III**	**TiO**_**2**_ **-NPs (5 mg/kg)**	**10.70 ± 2.23** ^**b**^	**25.64 ± 1.22** ^**b**^	**2.81 ± 0.57** ^**b**^
**IV**	**Acrylamide + TiO**_**2**_ **-NPs**	**12.11 ± 0.15** ^**b**^	**28.30 ± 0.90** ^**c**^	**3.55 ± 0.10** ^**c**^
**One Way Analysis of Variance**		**F = 24.83** *P* < 0.001	**F = 65.02** *P* < 0.001	**F = 38.29 P < 0.001**

• Six mice were used for each group.

• Results are expressed as mean ± SD.

• Results were analyzed using one-way analysis of variance followed by Duncan’s test to test the similarity between the control and three treated groups.

• According to Duncan’s test means with different letters indicates statistical significant difference between the compared groups in the same column at a significant level of *p* < 0.001.

### Overexpression of p53, INOS and COX-2 genes

As displayed in Table 3, oral administration of acrylamide (3 mg/kg b.w) or TiO_2_ nanoparticles (5 mg/kg b.w) separately, five times a week for two successive weeks, significantly (*p* < 0.001) increased the expression level of hepatic p53, INOS and COX-2 genes compared to those in the hepatic tissue of the negative control group. Moreover, simultaneous coadministration of acrylamide with TiO_2_ nanoparticles significantly (*p* < 0.001) elevated the expression level of these genes compared to their expression level in mice orally given acrylamide or TiO_2_ nanoparticles alone (Table 3).

**Table 3:** Expression level of p53, INOS, HO-1 and COX-2 genes in the hepatic tissues of the negative control group and groups given orally TiO_2_ nanoparticles (TiO_2_-NPs) or/and acrylamide.

Group	Treatment (dose mg/kg)	P53	INOS	HO-1	COX-2
I	Negative control (deionized water)	1.00 ± 0.00 ^a^	1.00 ± 0.00 ^a^	1.00 ± 0.00 ^a^	1.00 ± 0.00 ^a^
II	Acrylamide (3 mg/kg)	2.45 ± 0.08 ^b^	5.36 ± 0.31 ^b^	0.79 ± 0.02 ^b^	2.36 ± 0.07 ^b^
**III**	**TiO**_**2**_ **-NPs (5 mg/kg)**	**3.90 ± 0.13** ^**c**^	**3.23 ± 0.33** ^**c**^	**0.62 ± 0.04** ^**c**^	**4.65 ± 0.22** ^**c**^
**IV**	**Acrylamide + TiO**_**2**_ **-NPs**	**7.57 ± 0.53** ^**d**^	**4.11 ± 0.11** ^**d**^	**0.50 ± 0.03** ^**d**^	**6.29 ± 0.28** ^**d**^
**One Way Analysis of Variance**		**F = 312.23** *P* < 0.001	**F = 100.22** *P* < 0.001	**F = 117.23 P < 0.001**	**F = 514.33 p < 0.001**

• Six mice were used for each group and Results are expressed as mean ± SD.

• Results were analyzed using one-way analysis of variance followed by Duncan’s test to test the similarity between the control and three treated groups.

•According to Duncan’s test means with different letters indicates statistical significant difference between the compared groups in the same column at a significant level of *p* < 0.001.

### Down-expression of HO-1 gene

As seen in Table 3, the expression level of hepatic HO-1 gene was significantly (*p* < 0.001) reduced following separate oral administration of acrylamide or TiO_2_ nanoparticles for two successive weeks compared the negative control group. Furthermore, simultaneous coadministration of acrylamide (3 mg/kg) with TiO_2_ nanoparticles (5 mg/kg), five times weekly for two consecutive weeks, resulted in a significant decrease in hepatic HO-1 gene expression compared the levels observed in mice treated with either acrylamide or TiO_2_ nanoparticles alone (Table 3).

### Over-generation of hepatic ROS

**Fig. 3:**
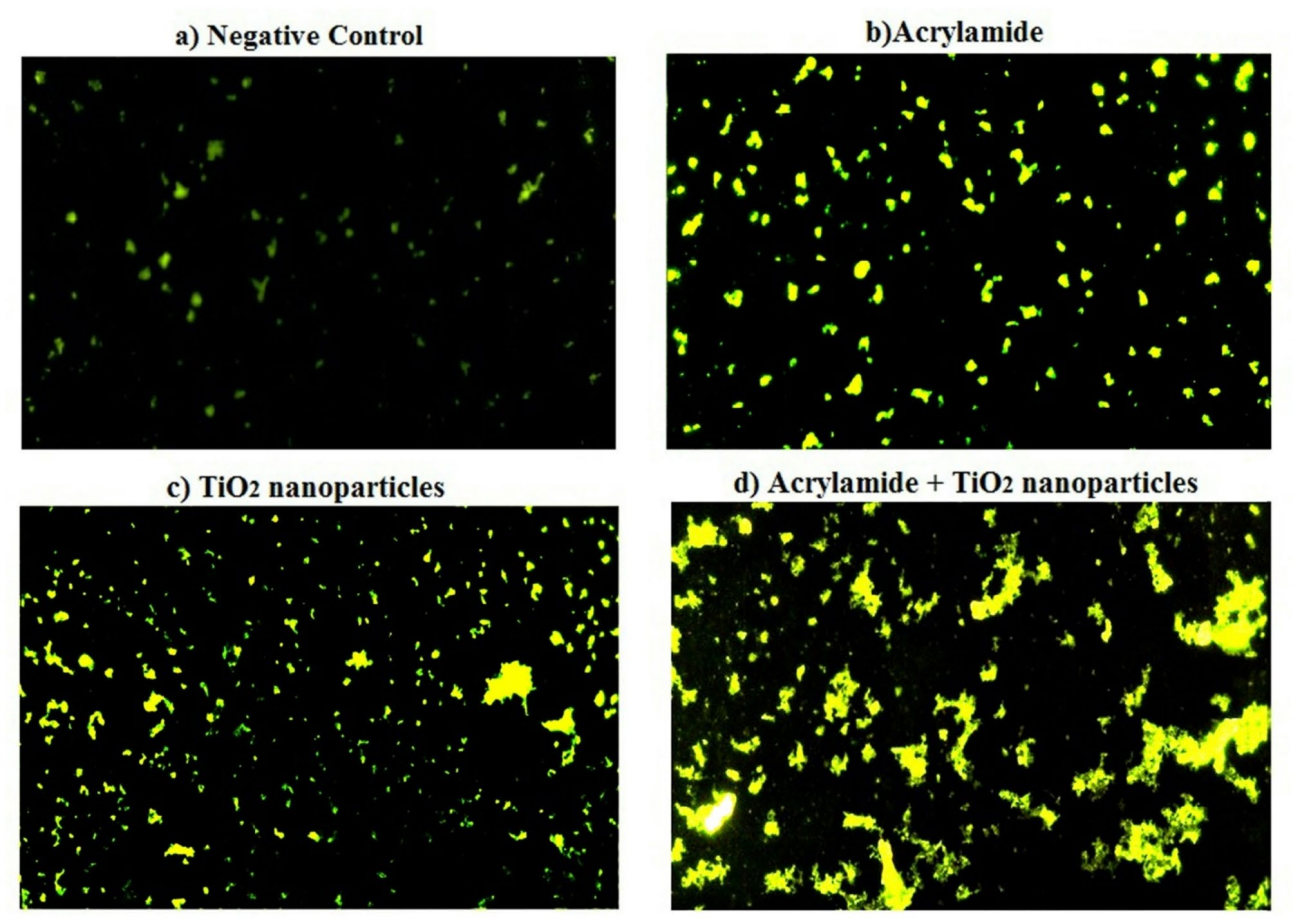
Level of ROS generation within the hepatic tissues of **a**) negative control group, **b**) acrylamide administrated group, **c**) TiO_2_ nanoparticles administrated group, and **d**) group administered acrylamide with TiO_2_ nanoparticles.

**Fig. 4:**
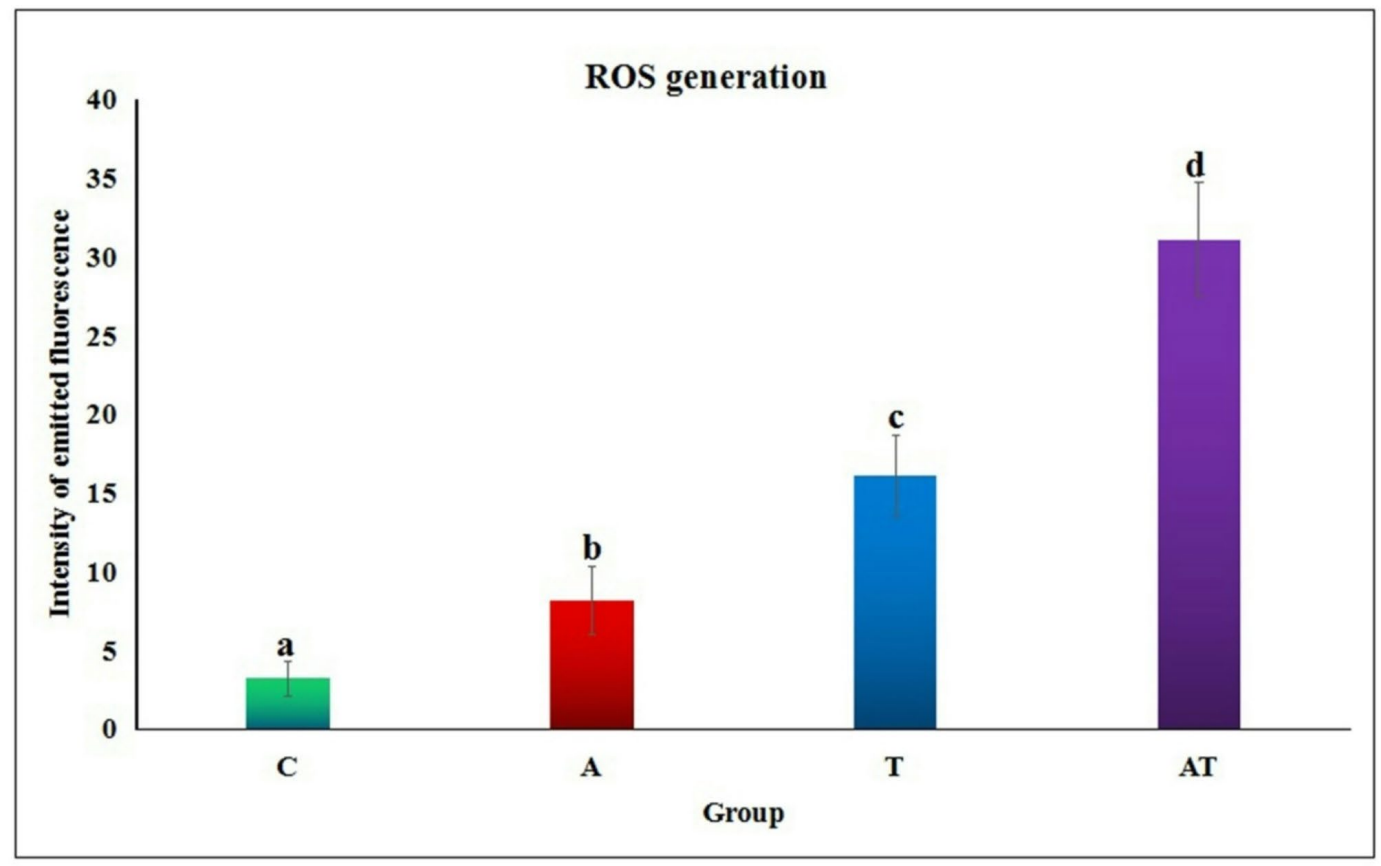
Quantity of ROS generated within the hepatic tissues of negative control group (C), acrylamide administrated group (A), TiO_2_ nanoparticles administrated group (T), and group administered acrylamide with TiO_2_ nanoparticles (AT). Results are expressed as mean ± SD. Different letters indicate statistical significant difference at *p* < 0.05.

Screening hepatocytes stained with 2, 7 dichlorofluorescin diacetate dye revealed significant increases in hepatic ROS generation following separate oral administration of acrylamide or TiO_2_ nanoparticles, as indicated by the significant increases (*p* < 0.001) in the intensity of emitted fluorescent light compared to the negative control cells (Figs. 3 and 4). Furthermore, simultaneous multiple oral coadministration of acrylamide with TiO_2_ nanoparticles also caused a significant elevation (*p* < 0.001) in hepatic ROS generation, evidenced by a substantial increase in fluorescent light intensity compared to hepatocytes from mice treated with either acrylamide or TiO_2_ nanoparticles alone (Figs. 3 and 4).

## Discussion

The rapid and widespread incorporation of TiO_2_ nanoparticles into everyday products such as chewing gum, toothpaste, cosmetics, moisturizers, and sweets has significantly increased human exposure to these particles. This exposure often occurs alongside other environmental and dietary contaminants, including acrylamide, heavy metals, and various food and water pollutants. Despite this growing concern, limited research has explored the combined effects of TiO_2_ nanoparticles with other toxicants on genomic DNA integrity, inflammation, and oxidative stress. To address this gap, the present study investigates the co-administration of acrylamide and TiO_2_ nanoparticles and their impact on DNA integrity, ROS production, and the expression of apoptotic and inflammatory genes in mouse liver tissue.

In this study, mice were orally administered acrylamide and TiO_2_ nanoparticles at doses of 3 mg/kg and 5 mg/kg, respectively. These doses were selected to reflect realistic human exposure levels, as individuals may ingest similar amounts through frequent consumption of contaminated food, beverages, and commercial products^4,26^. The results of this study demonstrated that chronic low-dose exposure to either acrylamide (3 mg/kg) or TiO_2_ nanoparticles (5 mg/kg) induced genotoxic effects. This was evidenced by significant increases in hepatic Comet assay parameters: tail length, %DNA in tail, and tail moment, compared to control values. Additionally, DNA laddering analysis revealed a smeared pattern of genomic DNA fragmentation in liver tissues of treated mice, confirming compromised genomic integrity. These findings align with earlier studies reporting the genotoxic potential of acrylamide and TiO_2_ nanoparticles, which include the induction of chromosomal aberrations, DNA strand breaks, and mutations across various biological systems^1,3,5,9,13–15,17^.

Despite the widespread use of acrylamide and TiO_2_ nanoparticles, their combined genotoxic effects on the liver have been largely unexplored, thereby necessitated studying the effect of acrylamide and TiO_2_ nanoparticles coadministration on hepatic genomic DNA integrity. Our results demonstrated that coadministration of acrylamide and TiO_2_ nanoparticles augments the genotoxicity induced by acrylamide or TiO_2_ nanoparticles alone through the significant increases observed in hepatic tail length, %DNA in tail and tail moment after acrylamide and TiO_2_ nanoparticles coadministration compared to their levels in mice orally given acrylamide or TiO_2_ nanoparticles alone. These results supported the recent studies that showed motivation and augmentation of genomic DNA damage induction after coadministration of acrylamide and TiO_2_ nanoparticles in brain and kidney tissues of mice^16,18^.

DNA damage serves as a crucial initiating factor that can result in genomic instability if left unrepaired. Various types of DNA lesions including strand breaks, base modifications, or crosslinks pose threats to the genome’s integrity. Normally, the cell’s DNA repair systems correct these defects; however, when DNA damage persists or is incorrectly repaired, it can lead to mutations, chromosomal rearrangements, and aneuploidy, all of which contribute to genomic instability^27^. Excessive DNA damage can overwhelm repair mechanisms, compromising genome maintenance and fostering the gradual buildup of genetic alterations that drive genomic instability^28^. Therefore, DNA damage acts as the initial catalyst for genomic instability by triggering mutagenic processes inducing widespread genomic changes.

Induction of oxidative stress and inflammation through excessive ROS generation is one of the acceptable mechanisms for acrylamide and TiO_2_ nanoparticles genotoxicity^5,9,16,18^. Induction of oxidative stress after administration of acrylamide or/and TiO_2_ nanoparticles through extensive hepatic ROS generation was manifested in the current study by the significant increases observed in the intensity of the emitted florescent light from hepatic cells stained with 2,7- dichlorofluorescin diacetate dye compared to that emitted from the stained negative control cells. As a result, coadministration of acrylamide and TiO_2_ nanoparticles caused marked increases in the hepatic ROS generation that impairs hepatocytes and makes it more susceptible to DNA damage induction because ROS are highly reactive and attack with cellular lipid, protein and DNA producing single- and double-DNA stranded breaks^29^.

The generation of extensive ROS and induction of DNA breaks also stimulates inflammation and cell death. For example, a single DNA break can cause chromosomal aberrations, homologous recombination, and mutations that disrupt the integrity of the genomic DNA and kill the cell^30–34^. Intensive ROS generation also disrupts the balance between oxidants and antioxidants and exhausts the antioxidant defense system leading to inflammation induction^35,36^. The induction of inflammation after repeated administration of low-dose acrylamide or/and TiO_2_ nanoparticles was mirrored through the noticed marked elevations in the expression level of the inflammatory COX-2 and INOS genes along with significant decreases in the expression level of the anti-inflammatory HO-1 gene compared to the negative control expression level. These findings are supported with the fact that INOS and COX-2 genes are highly expressed under inflammation producing massive amounts of pro-inflammatory and cytotoxic nitric oxide and prostaglandins that subsequently attack and inhibit the expression of the anti-oxidant and anti-inflammatory HO-1 gene^37^. Ongoing with our above-mentioned data, the augmentation of hepatic genomic DNA damage noticed after coadministration of acrylamide and TiO_2_ nanoparticles could be attributed to the observed significant upregulation of inflammatory COX-2 and INOS genes’ expression and the marked reductions in the anti-inflammatory HO-1 gene expression that weakens hepatic cells and makes them more susceptible to inflammation and apoptotic DNA damage induction compared to their expression level in mice orally given acrylamide or TiO_2_ nanoparticles alone.

Excessive DNA damage and ROS generation stimulate inappropriate apoptosis^38^. Apoptosis induction was reflected in our study through the significant upregulation of apoptotic p53 gene expression noticed after multiple administration of acrylamide or/and TiO_2_ nanoparticles compared to the negative control expression level. The expression level of hepatic p53 gene expression was also significantly elevated after chronic coadministration of acrylamide and TiO_2_ nanoparticles together which augmented acrylamide or TiO_2_ nanoparticles induced apoptotic DNA damage because overexpression of disrupts various cellular processes, induces genomic instability and triggers apoptosis^39,40^.

This study offers novel insights by being one of the first to investigate the combined genotoxic effects of acrylamide TiO_2_ nanoparticles on hepatic genomic DNA integrity in vivo, revealing that coadministration significantly amplifies DNA damage compared to individual exposures. The use of comet assay parameters provides quantitative evidence of this synergistic effect, highlighting the potential health risks of simultaneous exposure to common environmental and dietary toxicants. However, the study has some limitations: it was conducted in mice, which may not fully reflect human responses; it assessed only short-term exposure, leaving long-term effects unaddressed; and it focused solely on DNA damage in liver tissue without examining other toxicity pathways or molecular mechanisms. Additionally, dose–response relationships and interactions at varying concentrations were not explored, underscoring the need for further research to fully understand the mechanisms and implications of co-exposure.

## Conclusion

Based on the data discussed, it can be concluded that chronic administration of acrylamide (3 mg/kg) and/or TiO_2_ nanoparticles (5 mg/kg) even at the low doses, disrupted the integrity of hepatic genomic DNA and triggered inflammation by increasing ROS generation and causing abnormal alterations in inflammatory and apoptotic gene expression. Moreover, the concurrent administration of low-dose acrylamide and TiO_2_ nanoparticles exacerbated the toxic effects, amplifying acrylamide- or TiO_2_ nanoparticles induced apoptotic DNA damage and inflammation. This was achieved through the enhancement of hepatic ROS generation and the upregulation of inflammatory and apoptotic gene expression, alongside the inhibition of the anti-inflammatory HO-1 gene expression. These combined effects weaken hepatic cells, making them more susceptible to the toxicities induced by acrylamide and TiO_2_ nanoparticles. Therefore, it is recommended to avoid the concurrent administration of acrylamide and TiO_2_ nanoparticles, as their combined use increases the risk of severe hepatic toxicity. These findings provide crucial evidence for environmental, drug, and health-related agencies to better regulate TiO_2_ nanoparticles and food pollutants like acrylamide in products intended for human consumption, raising awareness of their potential harmful effects on human health.

## Supplementary Information

Below is the link to the electronic supplementary material.

Supplementary file1

## Data Availability

The datasets used and/or analyzed during the current study are available from the corresponding author on reasonable request.
