# Supplementary figures and images for: Acrylamide coadministration modulates hepatic ROS-mediated apoptotic DNA damage and inflammation induced by TiO2 nanoparticles in mice
Source: Sci Rep. 2025 Jul 15;15:25444. doi: 10.1038/s41598-025-10915-0 (PMC12259947; doi:10.1038/s41598-025-10915-0)

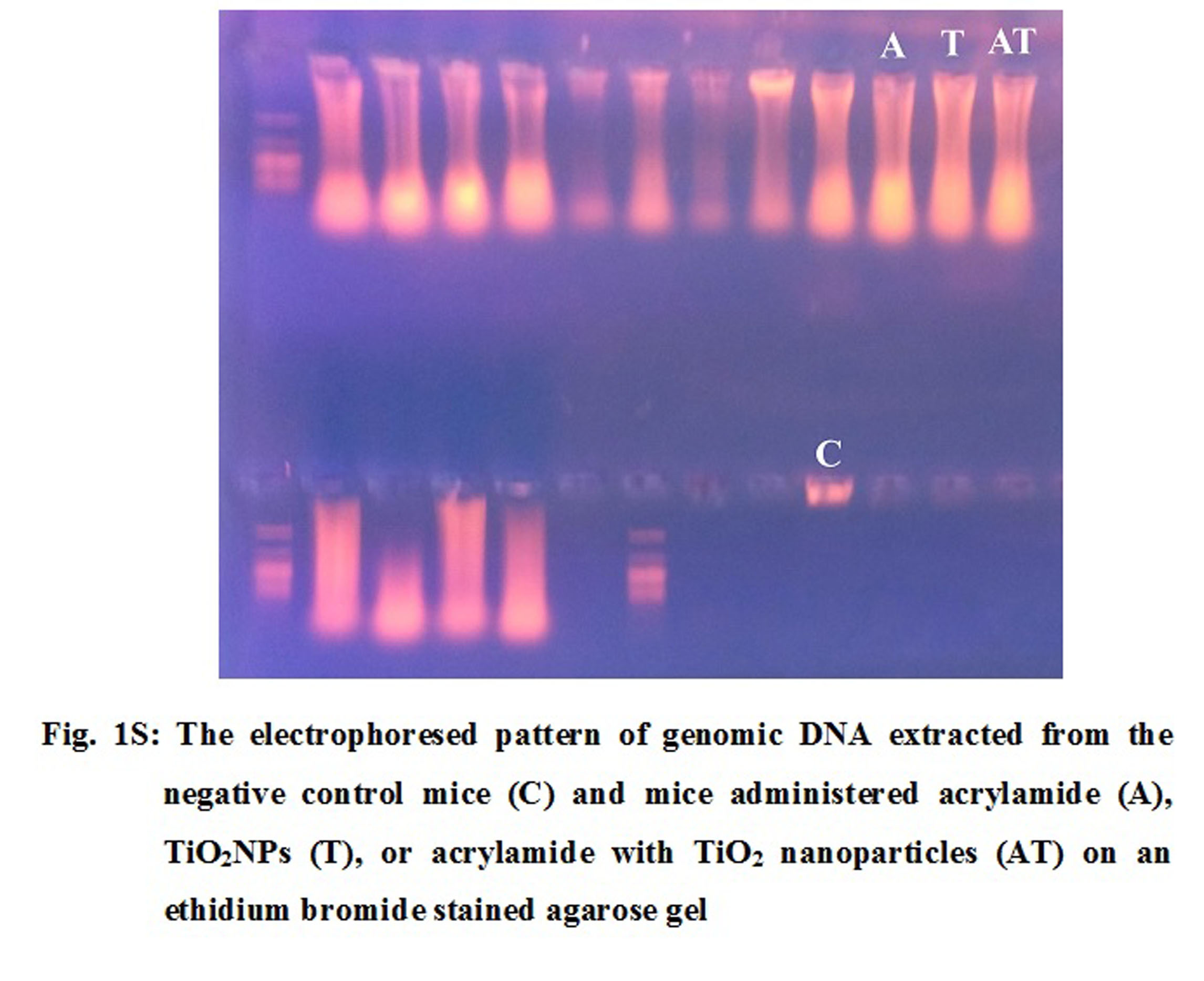

Supplement: Supplementary file 1 — Supplementary file1 [file 41598_2025_10915_MOESM1_ESM.jpg]
